# Sero-epidemiological survey and risk factors associated with bovine brucellosis among slaughtered cattle in Nigeria

**DOI:** 10.4102/ojvr.v83i1.1002

**Published:** 2016-05-12

**Authors:** Victor O. Akinseye, Hezekiah K. Adesokan, Akwoba J. Ogugua, Folashade J. Adedoyin, Patricia I. Otu, Ayi V. Kwaghe, Noah O. Kolawole, Oyinye J. Okoro, Charity A. Agada, Adeniyi O. Tade, Olufemi O. Faleke, Anyanwu L. Okeke, Ibikunle M. Akanbi, Mofoluwake M. Ibitoye, Morenike O. Dipeolu, Emma J. Dale, Perrett Lorraine, Andrew V. Taylor, Emmanuel A. Awosanya, Eniola O. Cadmus, Judy A. Stack, Simeon I. Cadmus

**Affiliations:** 1 Department of Veterinary Public Health and Preventive Medicine, University of Ibadan, Nigeria; 2 Department of Veterinary Medicine, University of Maiduguri, Nigeria; 3 Department of Veterinary Public Health and Preventive Medicine, University of Nigeria, Nigeria; 4 Department of Veterinary Public Health and Preventive Medicine, University of Agriculture, Nigeria; 5 Department of Veterinary Public Health and Reproduction, Federal University of Agriculture, Nigeria; 6 Department of Veterinary Public Health and Preventive Medicine, Uthman dan Fodiyo University, Nigeria; 7 National Veterinary Research Institute Vom, Plateau State, Nigeria; 8 Department of Veterinary Services, Ministry of Agriculture and Rural Development, Oyo State, Nigeria; 9 Department of Bacteriology and TB, Animal & Plant Health Agency, United Kingdom; 10 Department of Preventive Medicine and Primary Care, University of Ibadan, Nigeria

## Abstract

Bovine brucellosis is endemic in Nigeria; however, limited data exist on nationwide studies and risk factors associated with the disease. Using a cross-sectional sero-epidemiological survey, we determined the prevalence of and risk factors for brucellosis in slaughtered cattle in three geographical regions of Nigeria. Serum samples from randomly selected unvaccinated cattle slaughtered over a period of 3 years (between December 2010 and September 2013) from northern, southern and south-western Nigeria were tested for antibodies to *Brucella abortus* using the Rose Bengal test. Data associated with risk factors of brucellosis were analysed by Stata Version 12. In all, 8105 cattle were screened. An overall seroprevalence of 3.9% (315/8105) was recorded by the Rose Bengal test, with 3.8%, 3.4% and 4.0% from the northern, southern and south-western regions, respectively. Bivariate analysis showed that cattle screened in northern Nigeria were less likely to be seropositive for antibodies to *Brucella* spp. than those from south-western Nigeria (odds ratio = 0.94; 95% confidence interval: 0.73–1.22). However, logistic regression analysis revealed that breed ( *p* = 0.04) and sex ( *p* £ 0.0001) of cattle were statistically significant for seropositivity to *Brucella* spp. The study found that brucellosis was endemic at a low prevalence among slaughtered cattle in Nigeria, with sex and breed of cattle being significant risk factors. Considering the public health implications of brucellosis, we advocate coordinated surveillance for the disease among diverse cattle populations in Nigeria, as is carried out in most developed countries.

## Introduction

Brucellosis is a disease responsible for serious economic losses in the livestock industry. It is a zoonotic disease causing morbidity in humans and thus constitutes an important public health problem globally (Dean *et al*. 2012). Besides the economic impact brucellosis has on livestock production, it is estimated that about 500 000 persons are also infected annually (Pappas *et al*. 2006). The disease has been eradicated in most developed countries through the implementation of several extensive control programmes. On the other hand, developing countries have continued to experience an increasing trend of the disease because of lack of resources and coordinated control programmes. Other major factors contributing to the disease in sub-Saharan Africa include increased pastoralism and transhumance and intensification of commercial livestock farms (Ducrotoy *et al*. 2014).

Bovine brucellosis caused mainly by *Brucella abortus* (and in some instances, where mixed farming is practiced, by *Brucella melitensis*) is widespread in Africa, where it remains one of the most important zoonotic diseases (Ducrotoy *et al*. 2014; Gameel *et al*. 1993; Marcotty *et al*. 2009), with prevalence ranging from 6.6% to 41.0% among countries in West and Central Africa (Akakpo 1987; Bayemi *et al*. 2009; Kubuafor, Awumbila & Akanmori 2000; Schelling *et al*. 2003). Although the prevalence of bovine brucellosis is high and variable in many African countries, surveillance across the continent is generally poor (Marcotty *et al*. 2009; Pappas *et al*. 2006). Previous reports attributed the persistence and varying prevalence of the disease to factors that included purchase of infected cattle from the market for replacement or upgrading, nature of the animal production system, demographic factors, regulatory issues, climate, deforestation and wildlife interaction (Avong 2000; Muma *et al*. 2007; Musa, Jahans & Fadalla 1990; OIE 2011).

Most importantly, brucellosis is a contagious disease that is spread via direct contact with aborted foetuses, vaginal fluids, placentae and placental fluid. Therefore, veterinarians, abattoir workers and other livestock keepers are at risk of infection. Transmission can also be through the consumption of unpasteurised milk and milk products from infected animals (Ibironke *et al*. 2008). Sadly, however, adequate health and safety measures are rarely observed in most developing countries, hence increasing the chances of zoonotic transmission (Swai & Schoonman 2009). However, the fact that most animals, irrespective of where they originate, end up at the slaughter slabs or abattoirs is very important, because, apart from screening live animals at the herd level, screening slaughtered cattle at the abattoirs is also invaluable for the epidemiological investigation of bovine brucellosis.

In Nigeria, available seroprevalence studies have shown that bovine brucellosis is endemic in the country (Ate *et al*. 2007; Cadmus *et al*. 2006; Ishola & Ogundipe 2000; Ocholi 1990). In addition, evidence abounds that the practice of transhumance (seasonal movement of people and livestock between summer and winter pastures) among the Fulani pastoralists (a tribal group that holds the majority of the cattle population in Nigeria) has facilitated the spread of the disease across Nigeria (Bale & Kumi-Diaka 1981; Gameel *et al*. 1993). Despite these, few broad-based epidemiological studies exist that provide empirical data on the burden and distribution of brucellosis among diverse cattle populations simultaneously across different geographical regions of Nigeria. Therefore, to fill this important epidemiological gap, we conducted a large survey over a period of 3 years to determine (1) the seroprevalence of brucellosis among slaughtered cattle across different regions of the country and (2) the risk factors associated with the disease.

## Materials and methods

### Study sites

**FIGURE 1:**
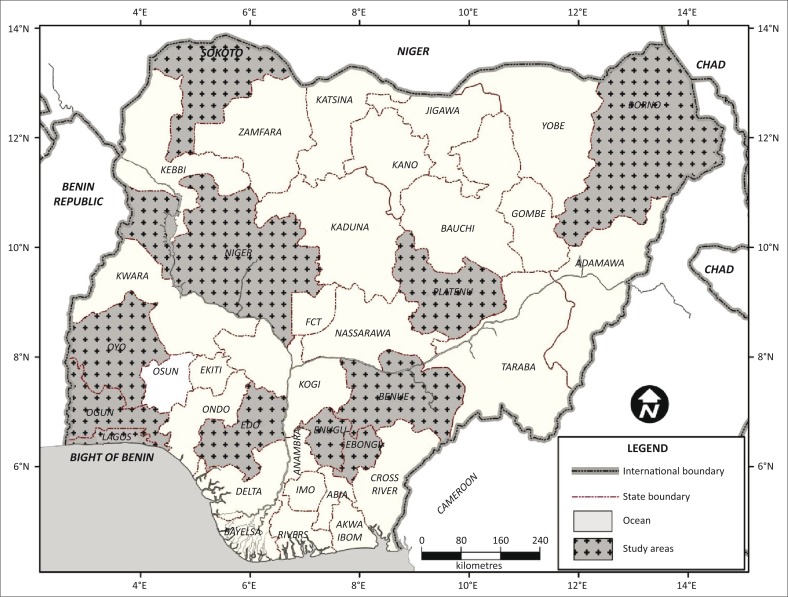
Map of Nigeria showing states in the regions where the study was conducted.

Nigeria has a population of over 170 million people and about 13 million cattle according to the National Population Census published in 2006 and the draft report of the National Agriculture Sampling Survey in 2011. The country is divided into six geographical regions of which three, namely northern, southern and south-western, were purposively selected for this study based on the slaughter cattle population in the areas. Nigeria is located in the West African subregion and bordered in the north by Niger Republic and Chad, in the south-west by Benin Republic and south-east by Cameroon (Figure 1). There is active transboundary movement of animals between Nigeria and neighbouring countries. Traditionally, agriculture including livestock farming is the mainstay of the people in Nigeria, and this is characterised mostly by close interactions between humans and animals.

#### Northern region

Five states including Benue, Borno, Niger, Plateau and Sokoto States (Figure 1) were chosen from the northern region. Borno and Sokoto States are among the highest cattle-producing states in Nigeria and also serve as a source of cattle to other parts of the country.

#### Southern region

Three states with major livestock activities, Ebonyi, Edo and Enugu States (Figure 1), were purposively selected in this region. Cattle slaughtered in these states originate from the northern region as well as neighbouring African countries.

#### South-western region

In the south-western region, Lagos, Ogun and Oyo States (Figure 1) were selected because they account for the highest volume of cattle slaughtered in Nigeria. In addition, cattle slaughtered in these states are sourced from northern Nigeria, neighbouring African countries and a few locally bred animals within the region.

### Duration of study, animal sampling, sample collection and handling

The study was carried out over a period of 3 years between December 2010 and September 2013. The major abattoir in each chosen state was purposively selected for animal sampling. Furthermore, all the abattoirs chosen receive their animal supply from diverse sources, which include: (1) local herds that are extensively managed, (2) trade cattle sourced from different markets within the state/region, (3) trade cattle sourced from different markets within northern Nigeria and (4) markets from neighbouring African countries. On average in each abattoir, blood samples were collected randomly from at least 5% – 10% (based on the volume of slaughter, which ranged from between 20 and 1000 cattle per day) of cattle slaughtered during the study period. At sampling, the breed, sex and age of all animals were recorded. The age of the animals were estimated into ≥ 2 years (adults) and < 2 years (young adults) using the teething method (Pace & Wakeman 2003). These age groups were chosen because the puberty period of two common breeds in Nigeria was estimated to be 19.0–23.5 months in Rahaji (Oyedipe *et al*. 1982) and 40.2 months in Sokoto Gudali (Knudsen & Sohael 1970). In addition, average body condition score of cattle slaughtered in each abattoir/state was documented as good, fair or poor (Nicholson & Butterworth 1986). When ≥ 70% of the animals slaughtered in a state were apparently healthy and well-fed, the overall body condition of animals slaughtered in the state was graded as good, when between 50% and 69% as fair and < 50% as poor (Table 1). Overall, all animals slaughtered were assumed to be unvaccinated against brucellosis because no programme for this exists in Nigeria nor within the neighbouring African countries where the animals were sourced (Ducrotoy *et al*. 2014).

**TABLE 1:** Distribution of cattle screened according to sex, age, breed and geographical regions in Nigeria.

Variables	Characteristic	Number of animals	Percent of animals
Sex	Male animals	4385	54.1
	Female animals	3720	45.9
Age	Adult	7541	93
	Young adult	564	7
Breed	Bunaji	4807	59.3
	Rahaji	2074	25.6
	Sokoto Gudali	444	5.5
	Mixed	410	5.1
	Adamawa Gudali	220	2.7
	Kuri	131	1.6
	Djali	19	0.2
Geographical regions	South-west	4667	57.6
	North	2356	29.1
	South	1082	13.4

**Total**	**-**	**8105**	**100**

In all, approximately 10 mL of blood was collected per animal in a sterile vacutainer tube during slaughter by a trained technician. Serum samples obtained from animals in each state for Rose Bengal test (RBT) were stored at -20 °C until they were transported under cool conditions and later assayed at the Tuberculosis and Brucellosis Laboratories of the Department of Veterinary Public Health and Preventive Medicine, University of Ibadan, Nigeria.

## Ethical consideration

The protocols for this study were approved by the University of Ibadan/University College Hospital Ethics Committee (NHRFC/05/01/2008a).

### Assay of samples

The serum samples were tested with RBT as described by Alton *et al*. (1988). The RBT antigen consisting of standardised *B. abortus* antigen (controls) was sourced from the Animal and Plant Health Agency, Surrey, UK. Briefly, equal volumes (30 μL) of antigen and test serum were mixed thoroughly on a plate using a stick applicator and the plate was rocked for 4 min. The appearance of agglutination within 1 minute was scored 2+ (++), whilst any agglutination between 1 and 4 min was scored 1+ (+). The absence of agglutination within 4 minutes of rocking was regarded as negative (−).

### Statistical analysis

Data analysis was carried out using Stata Version 12. Group differences were tested using chi-square statistics for categorical variables. A multivariable logistic regression was carried out using all the variables that were statistically significant at the 10% level with the main outcome measure (RBT) in bivariate analysis. All tests were two-tailed and statistical significance was set at *p* < 0.05.

## Results

In all, 8105 cattle were sampled from 11 states in the three geographical regions of Nigeria. About two-thirds (59.3%) of the animals were of the Bunaji breed, over half were male animals (54.1%) and the majority were adult (93.0%) (Table 1). Overall, the general body condition and physical health status of cattle slaughtered in the south-western, southern and northern regions were observed to be poor, fair and good, respectively (Table 2). A seroprevalence of 3.9% was obtained by RBT (Table 3). Overall, the highest (4.0%) seroprevalence was recorded in the south-western region, followed by the northern (3.8%) and the lowest (3.4%) from the southern region (Table 3). Generally, the breed-specific result showed that the highest seroprevalence occurred amongst the mixed-breed cattle (6.1%), followed by Rahaji (4.6%), other breeds (4.6%), Bunaji (3.5%) and Sokoto Gudali (1.8%) breeds of cattle, respectively (Table 3). Higher age-specific seroprevalence was recorded in the adults (≥ 2 years) (3.9%) when compared to the young adults (< 2 years) (3.2%). In addition, the sex–specific result showed higher seroprevalence among the female (4.7%) than the male (3.2%) cattle (Table 3).

**TABLE 2:** Proportion of healthy cattle in good condition slaughtered based on states and geographical regions in Nigeria.

States in Nigeria	City in Nigeria	Proportion (%)^a^
Northern	Sokoto	≥ 70
	Borno	60
	Plateau	≥ 70
	Niger	≥ 70
	Makurdi	≥ 70
Southern	Enugu	50–69
	Ebonyi	50–69
	Edo	≥ 70
South-western	Ibadan	< 50
	Lagos	≥ 70
	Ogun	< 50

a This was calculated based on the observed number of healthy versus emaciated animals slaughtered in each abattoir under study in the respective state.

**TABLE 3:** Brucellosis seroprevalence in cattle slaughtered according to sex, age, breed and geographical region in Nigeria using the Rose Bengal test.

Variables	Characteristic	Seropositive animals based on RBT				Odds ratio	95% CI	*p*-value

		Positive, *n*	%	Negative, *n*	%			
Sex	Male animals	140	3.2	4245	96.8	1	-	-
	Female animals	175	4.7	3545	95.3	1.47	1.16–1.88	0.002
Age	Adult	296	3.9	7245	96.1	1	-	-
	Young adult	19	3.2	545	96.6	0.87	0.54–1.39	0.553
Breed	Sokoto Gudali	8	1.8	436	98.2	1	-	-
	Rahaji	96	4.6	1978	95.4	2.6	1.28–5.48	0.01
	Bunaji	169	3.5	4638	96.5	2.0	0.97–4.06	0.080
	Mixed	25	6.1	385	93.9	3.5	1.58–7.94	0.002
	Others^a^	17	4.6	353	95.4	2.6	1.12–6.15	0.036
Geographical region	South-western	188	4.0	4479	96.0	1	-	-
	Northern	90	3.8	2266	96.2	0.94	0.73–1.22	0.67
	Southern	37	3.4	1045	96.6	0.84	0.58–1.21	0.84

RBT, Rose Bengal test.

a Kuri, Adamawa Gudali and Djali breeds were grouped into others for statistical analysis because they contained cells whose numbers were less than 5.

The results of the bivariate analysis showed that the breed ( *p* = 0.04) and sex ( *p* = 0.002) were significant factors associated with seropositivity of cattle for antibodies to *Brucella* spp. In addition, cattle from the northern region showed less likelihood of being seropositive for antibodies to *Brucella* spp. when compared to those from the south-western region [odds ratio (OR) = 0.9; 95% confidence interval (CI): 0.73–1.22; Table 3]. Furthermore, our results revealed that female cattle were more likely to be seropositive in comparison to the male cattle (OR = 1.5; 95% CI: 1.16–1.88), whilst the mixed-breed cattle were more likely to be seropositive than the Bunaji breed (OR = 1.8; 95% CI: 1.17–2.81; Table 4).

**TABLE 4:** Results of logistic regression analysis of variables significant at 10% level with the main outcome measure (RBT) in bivariate analysis.

Variable	Odds ratio	95% CI	*p*-value
**Sex**	**-**	**-**	**-**
Male animals	1.0 (referent group)	-	-
Female animals	1.46	1.16–1.83	0.00
**Breed**	**-**	**-**	**-**
Sokoto Gudali	1.0 (referent group)	-	-
Rahaji	2.6	1.27–5.48	0.01
Bunaji	1.9	0.97–4.06	0.06
Mixed	3.5	1.57–7.93	0.00
Others^a^	2.6	1.11–6.15	0.03

a Kuri, Adamawa Gudali and Djali breeds were grouped into others for statistical analysis because they contained cells whose numbers were less than 5.

## Discussion

A large sero-epidemiological survey of brucellosis was conducted among slaughtered cattle in Nigeria, revealing an overall seroprevalence of 3.9%. It was observed that cattle screened in the northern and southern regions of Nigeria were about 0.9 and 0.8 times less likely to be seropositive to *Brucella* infection than those from the south-western region. Despite the varying seroprevalence of bovine brucellosis found across the country, the findings indicated that the disease had spread among cattle that were slaughtered, hence reiterating its endemicity (although low) in Nigeria. This has far-reaching public health implications considering the consumption of unpasteurised milk, lack of personal protective equipment by butchers and other risk practices among livestock workers in Nigeria (Ibironke *et al*. 2008).

Findings from this study are corroborated by an earlier study carried out in central Oromiya, Ethiopia, where variations were observed in the seroprevalence of bovine brucellosis among cattle in the agro-ecological areas studied (Jergefa *et al*. 2009), in different countries in West Africa (Unger *et al*. 2003), and in small ruminants in different regions in Ethiopia (Teshale *et al*. 2006). However, the overall seroprevalence of 3.9% obtained in this study is lower than the 8.6% (Cadmus *et al*. 2013) and 5.8% (Cadmus *et al*. 2006) previously reported in south-western Nigeria. Although it is lower than the 12% from slaughtered cattle in Tanzania reported by Swai and Schoonman (2009), it is comparable to the findings of investigators from other developing countries: 4.9% in slaughtered cattle in Cameroon (Shey-Njila *et al*. 2005), 3.2% (Berhe, Belihu & Asfaw 2007) and 3.5% (Megersa *et al*. 2011) in Ethiopia, 3.3% in Central Africa (Nakouné *et al*. 2004) and 4.2% in Eritrea (Omer *et al*. 2000). The lower prevalence obtained in this study could be attributed to the fact that earlier studies in Nigeria were localised to specific areas, in contrast to the widespread coverage in this study. Therefore, the wider coverage and longer duration of this study might have had a diluting effect on the overall seroprevalence obtained.

Again, our findings show that cattle screened in northern and southern Nigeria are less likely to be seropositive to antibodies to *Brucella* species than compared to those from the south-western region (OR = 0.9; 95% CI: 0.73–1.22) (Table 3). This finding may be attributed to the fact that cattle herds in northern Nigeria are less likely to be infected with *Brucella* because of the effect of the persistent scorching sun in the area because the organism is less likely to survive in the environment under high temperatures (Ducrotoy *et al*. 2014).

The body condition of cattle screened could also be a contributing factor to the differences in seroprevalence observed across the geographical regions studied. Fewer than 50% of the animals slaughtered in the south-west were apparently physically healthy and well-fed compared with ≥ 70% from the northern region. Because body score provides an indication of the total health status of an animal (Nicholson & Butterworth 1986), it is plausible to infer that cattle slaughtered in south-western Nigeria are more likely to be infected with brucellosis. Our finding, though contrary to the assertion made by Wadood *et al*. (2009), is supported by the report of Kebede, Ejeta and Ameni (2008), who observed an increasing seroprevalence in animals with good, medium and poor body condition in a similar study conducted in Ethiopia. Furthermore, the Fulanis do not often sell off animals, particularly the female cattle, unless they are sick and unproductive or when they are in serious need of funds. This practice of the Fulanis was also reported in Togo (Dean *et al*. 2013). Considering the results, one can infer that most of the unhealthy and emaciated animals that are sold cheaply end up in markets and abattoirs where traders have lower purchasing power. In south-western Nigeria, more of the rich traders are concentrated in Lagos, hence the slaughter of more healthy cattle, as opposed to Ogun and Oyo States that slaughter a larger population of sick animals (Table 2). According to this scenario, one can safely infer that the epidemiology of brucellosis in slaughtered cattle in Nigeria is also informed by the purchasing power of stakeholders in the industry.

The difference in the breed-specific prevalence is in line with the findings of other researchers who have shown that breed was associated with brucellosis in cattle in Nigeria (Cadmus, Adesokan & Stack 2008; Cadmus *et al*. 2013; Junaidu, Oboegbulem & Salihu 2011; Mai, Irons & Thompson 2012). This is also consistent with the reports by Kubuafor *et al*. (2000) in Ghana, Karimuribo *et al*. (2007) in Tanzania and Matope *et al*. (2011), who associated the proportion of seropositive animals to breeds. Our findings show that mixed breed (mostly crosses between Rahaji and Bunaji) had the highest seroprevalence. As reported earlier, genetic variation is an important factor in conferring resistance or tolerance of cattle breeds to diseases, whilst the antibody response of animals resistant to infection by *B. abortus* differed significantly from those of susceptible ones (Martínez *et al*. 2010).

The findings of this study also show that female cattle were more likely to be seropositive to antibodies to *Brucella* spp. than male cattle (OR = 1.5; 95% CI: 1.16–1.88). These results, though contrary to the reports by Cadmus *et al*. (2013), are consistent with other reports that showed significantly higher seroprevalence in the female than male cattle (Bekele *et al*. 2000; Dinka & Chala 2009; Junaidu *et al*. 2011; Kebede *et al*. 2008; Kubuafor *et al*. 2000; Megersa *et al*. 2011; Tolosa *et al*., 2008). Generally, female animals are kept for an extended period of time providing a longer time of exposure to the pathogen, which could in turn serve as a source of infection for other animals. Moreover, female cattle are only culled when there is reduced reproductive performance or as a result of old age, at which time their risk of exposure would be high. In addition, the stress associated with pregnancy as well as calving, which tends to reduce immunity of female animals, may also explain the higher seroprevalence among female animals in this study. Although this factor was not considered at the inception of the study, it is an important consideration for future studies because some of the female animals slaughtered during this study were pregnant.

Age did not play any significant role in seropositivity of cattle for antibodies to *Brucella* spp. in this study. This is contrary to the normal pattern of brucellosis spread in a cattle population reported previously (Berhe *et al*. 2007; Mai *et al*. 2012; Matope *et al*. 2011). However, it has been reported that young animals infected *in utero* could be latently infected, only to show evidence of infection in later years (Hinic *et al*. 2009; Nielsen 2000; Robinson 2003). This finding may also be understandable because the cattle population screened were not from conventional herds but diverse cattle populations and mostly trade cattle. Nonetheless, our finding agrees with that of Jergefa *et al*. (2009), who showed no significant association between the age of cattle and seropositivity to *Brucella* antibodies. The current findings could be attributed to the varying proportions of adult to young cattle that were screened (more than ten times), which is similar to more adult animals sampled by Jergefa *et al*. (2009). Again, we suspect that a sizeable proportion of the young cattle in our study could be from infected dams (a likely reason why they were sold), thus increasing the overall prevalence of brucellosis in the population against what is generally observed (Bayemi *et al*. 2009).

This study had some limitations. Firstly, the use of purposive sampling resulted in sampling of more animals in south-western Nigeria compared to other regions. The reason is that more animals destined for slaughter in Nigeria are found in south-western Nigeria, particularly Lagos State (with the highest human population compared to other states in the country). This also necessitated more active routine screening of slaughtered cattle for brucellosis in south-western Nigeria. The authors, however, believe that this difference did not significantly affect the results of this study, given the use of proportions in determining the relative prevalence of the disease.

Secondly, RBT was used as the screening tool in this study, as with many other brucellosis seroprevalence studies in Africa (Matope *et al*. 2011). Its simplicity and relatively low cost (McGiven 2013) are important considerations in low-resource settings like Nigeria, where vaccination of cattle is seldom carried out. However, it is accepted that false-positive results because of cross-reactions may be obtained and that confirmation of positive results is recommended (OIE 2011). Lastly, bacteriological isolation of *Brucella* spp. was not performed; this could have helped to confirm the species of *Brucella* circulating among the cattle population screened and provided a better insight into the epidemiology of the disease. However, earlier studies in Nigeria, other developing and developed countries, have found serological investigation useful for large-scale studies similar to this one.

## Conclusion

It was verified that bovine brucellosis is endemic in Nigeria. Higher seroprevalence was observed in the south-western compared to southern and northern regions, with the odds of brucellosis seropositivity lower in other regions compared to those in south-western Nigeria. Furthermore, our findings show that breed and sex of cattle play significant roles in the epidemiology of brucellosis in cattle population in Nigeria. Based on these findings, we suggest that more diverse epidemiological contexts (e.g. management systems, trade and transhumance systems, agro-ecological zones and climatic conditions) be studied across the country in order to provide a better platform for informed control measures to mitigate challenges peculiar to each region and the country at large. Finally, we advocate for coordinated research to determine various social drivers responsible for the epidemiology of brucellosis in Nigeria.
